# NONVERBAL STRATEGIES TO ENHANCE PERSON-CENTERED COMMUNICATION WITH PEOPLE LIVING WITH DEMENTIA

**DOI:** 10.1093/geroni/igac059.1874

**Published:** 2022-12-20

**Authors:** Emma Bender, Marie Savundranayagam, Laura Murray, Joseph Orange

**Affiliations:** Western University, London, Ontario, Canada; Western University, London, Ontario, Canada; Western University, London, Ontario, Canada; Western University, London, Ontario, Canada

## Abstract

Many people living with dementia experience difficulties comprehending language, and benefit from nonverbal communication (NVC). Yet, little published empirical evidence exists for care partners regarding NVC strategies that support person-centered communication with perons living with dementia. This study aimed to determine whether NVC strategies used by personal support workers accompany verbal communication demonstrating person-centered communication indicators (facilitation, negotiation, recognition, validation). Secondary data analysis of video-recorded interactions (n=40) between personal support workers and simulated persons living with dementia was conducted. The recordings were transcribed according to communication-units, which were coded for person-centered communication and NVC, using a novel coding system consisting of ten NVC strategies. The overlap between NVC strategies and verbal person-centered communication was examined. Findings revealed that personal support workers frequently accompany verbal person-centered communication with NVC strategies. Out of 1848 communication-units in which person-centered verbal communication was used, 69% overlapped with NVC strategies. Gaze overlapped with all person-centered communication indicators frequently, both individually (40% – 49% of overlapping communication-units) and when combined with touch (13-24%). Gestures using objects (with and without gaze) frequently accompanied facilitation (17%) and negotiation (21%), while positive facial expressions (with and without gaze) were commonly found in recognition (16%) and validation (16%). The use of NVC strategies which support person-centered communication may lead to communication enhancement, in turn improving interactions and relationships between persons living with dementia and their care partners.

